# Integrative identification of key genes governing *Verticillium* wilt resistance in *Gossypium hirsutum* using machine learning and WGCNA

**DOI:** 10.3389/fpls.2025.1621604

**Published:** 2025-07-28

**Authors:** Yufeng Lei, Jing Zhao, Siyuan Hou, Fufeng Xu, Chongbo Zhang, Dongchen Cai, Xiaolei Cao, Zhaoqun Yao, Sifeng Zhao

**Affiliations:** ^1^ Key Laboratory at the Universities of Xinjiang Uygur Autonomous Region for Oasis Agricultural Pest Management and Plant Protection Resource Utilization, Agriculture College, Shihezi University, Shihezi, China; ^2^ Cotton Research Institute, Xinjiang Academy of Agricultural and Reclamation Sciences, Shihezi, China

**Keywords:** *Verticillium* wilt, *Gossypium hirsutum*, RNA-Seq, WGCNA, machine learning, disease resistance

## Abstract

**Introduction:**

*Verticillium* wilt, caused by *Verticillium dahliae*, is one of the most devastating diseases affecting global cotton (*Gossypium hirsutum*) production. Given the limited effectiveness of chemical control measures and the polygenic nature of resistance, elucidating the key genetic determinants is imperative for the development of resistant cultivars. In this study, we aimed to dissect the temporal transcriptional dynamics and regulatory mechanisms underlying *Gossypium hirsutum* response to *V. dahliae* infection.

**Methods:**

We employed a time-course RNA-Seq approach using the susceptible upland cotton cultivar Jimian 11 to profile transcriptomic responses in root and leaf tissues post-*V. dahliae* inoculation. Differentially expressed genes (DEGs) were identified, followed by weighted gene co-expression network analysis (WGCNA). To prioritize key candidate genes, we applied machine learning algorithms including LASSO, Random Forest, and Support Vector Machine (SVM).

**Results and discussion:**

A robust set of core genes involved in pathogen recognition (*GhRLP6*), calcium signaling (*GhCIPK6, GhCBP60A*), hormone response, and secondary metabolism (*GhF3’H*) were identified. Our findings provide novel insights into the spatiotemporal regulation of immune responses in cotton and offer valuable candidate genes for molecular breeding of *Verticillium* wilt resistance.

## 1 Introduction

Cotton (*Gossypium* spp.) is an economically and globally significant fiber and agricultural crop, threatened severely by *Verticillium* wilt (Zhang et al., 2022). Cotton *Verticillium* wilt, caused by *Verticillium dahliae*, is now one of the significant barriers to sustainable Chinese cotton yields (Zhang et al., 2025b). *V. dahliae* persists in soil as microsclerotia that can live longer than 14 years (Short et al., 2015). The microsclerotia germinate to form infectious hyphae that infect cotton plants from roots and infect the vasculature of plants when provided with proper growth surroundings, leading to common signs of leaf chlorosis, wilting, defoliation, and whole-plant death (Short et al., 2015). Due to the structural durability of microsclerotia and *V. dahliae*’s varied races, *Verticillium* wilt is highly challenging to control. Now, there is no highly effective fungicide in chemical control available, and production of disease-resistant varieties is considered to be the best economic and effective measure to control the pathogen (Zhang et al., 2025c).

Recently, with advancements in molecular breeding, scientists have used marker-assisted selection (MAS) to map numerous quantitative trait loci (QTL) of Verticillium wilt resistance in cotton and steadily deciphered the mechanisms of resistance (Ayyaz et al., 2025; Khan et al., 2025). The NBS-LRR type resistance gene GbaNA1 in *Gossypium barbadense* and TIR-NBS-LRR gene GhDSC1 in *G. hirsutum* have been reported to strongly advance the resistance of plants to *V. dahliae* by mediating effector-triggered immunity (ETI) (Li et al., 2018; Li et al., 2019). The receptor kinase-mediated pattern recognition receptor (PRR) pathways also play significant roles in cotton resistance to disease. As examples, the receptor-like kinase gene *GhRLCK*-VII and the wall-associated kinase gene *GhWAK7A* render resistance by, respectively, recognizing pathogen-associated molecular patterns (PAMPs) and cooperatively activating downstream immune cascades, thereby mediating the primary PAMP-triggered immunity (PTI) response in cotton (Liu et al., 2023; Wang et al., 2020). The secondary metabolites’ accumulation offers an essential chemical defense to cotton as well; as examples, phenylpropanoid and flavonoid secondary metabolic pathways are intimately related to improved antimicrobial ability (Xiao et al., 2023, 2021). Generally speaking, cotton resistance to *Verticillium* wilt is highly complex and entailed by numerous genes and hormone signaling as well as secondary metabolism, whereas resistance mechanisms of elite resistant germplasm remain to be fully elucidated.

In past few years, RNA-seq has been widely applied to study cotton disease resistance mechanisms. Transcriptomic analysis enables genome-wide monitoring of gene expression changes in cotton after *V. dahliae* infection, facilitating the discovery of resistance-related pathways and key genes. For example, one study used time-series transcriptomics combined with weighted gene co-expression network analysis (WGCNA) to construct a gene regulatory network of cotton’s response to *Verticillium* wilt and identified core resistance gene modules (Yang et al., 2024). In this study, we used a *Verticillium* wilt-susceptible upland cotton cultivar, Jimian 11, inoculated it with *V. dahliae* and collected samples at multiple time points, and performed transcriptome sequencing to dissect the dynamic transcriptional regulatory mechanisms of cotton’s defense response. Meanwhile, we integrated differential expression analysis, WGCNA co-expression network analysis, and machine learning to identify key genes (Zhang et al., 2024). Our study deepens the understanding of the molecular mechanisms of cotton resistance to *Verticillium* wilt and provides candidate genes and a theoretical basis for molecular breeding of disease-resistant varieties.

## 2 Materials and methods

### 2.1 Plant materials and pathogen inoculation

The experimental cotton was *G. hirsutum* cultivar Jimian 11. Cotton seeds were surface-sterilized and germinated in Petri dishes. When the radicle grew to 3–5 cm, seedlings were transferred to Hoagland nutrient solution for hydroponic cultivation. *V. dahliae* (The strain was a highly virulent isolate collected from a *Verticillium* wilt-infected cotton) was first cultured on PDA medium for 7 days. A mycelial plug was then transferred to liquid medium and agitated at 200 rpm and 26 °C for 3 days. The mycelial mat was passed through gauze, and the resulting spore suspension was diluted to 1×10^6^ spores/mL. This suspension was introduced into the cotton hydroponic boxes for inoculation. After 12 h of incubation, the spore suspension was removed and replaced with sterilized water. Leaf and root samples were collected at 0 h, 12 h, 24 h, and 48 h post inoculation. All samples were immediately frozen in liquid nitrogen and stored at –80°C until use.

### 2.2 Sampling and RNA-seq

For each time point and tissue, three individual plants were sampled as biological replicates, yielding 24 samples total (2 tissues × 4 time points × 3 replicates). Total RNA was isolated in every leaf and root sample via Trizol method. The integrity and purity of RNA were verified using NanoDrop spectrophotometry and agarose gel electrophoresis. The RNA of each sample was applied to construct libraries for sequencing: mRNA was enriched and afterwards fragmented, first-strand cDNA was synthesized, adapters were ligated, and PCR amplification was done to achieve the final library. All libraries were sequenced following library quality control onto an Illumina HiSeq X platform. Library construction and sequencing were performed by Novogene Co., Ltd. Raw sequencing reads were processed with Trimmomatic (v0.39) to remove adapters and low-quality reads and evaluated with FastQC (v0.12.1) for quality control, yielding high-quality clean reads. The clean reads were aligned to the cotton reference genome (*G. hirsutum* (AD1) ‘TM-1’ T2T genome JZU_v1.0 from the CottonGen database, Yan et al., 2025) using HISAT2 (v2.2.1). Transcript abundance was quantified as transcripts per million (TPM) using Salmon (v1.9.0).

### 2.3 Differential expression analysis and GO enrichment

Differentially expressed genes (DEGs) were identified using the DESeq2 v1.48.1R package (Love et al., 2014). The 0 h samples were used as control, gene expression at each treatment time point (12 h, 24 h, 48 h) was compared for both leaves and roots. DEGs were determined through DESeq2 R package (Love et al., 2014). The 0 h samples served as control, and gene expression at every treatment duration (12 h, 24 h, 48 h) was contrasted in both roots and leaves. DEGs were identified by using |log_2_ (Fold Change) | ≥ 1 and FDR < 0.05 as the threshold. Gene Ontology (GO) enrichment analysis of the identified differentially expressed genes (DEGs) was performed using the R package clusterProfiler (v4.16.0; Wu et al., 2021). The *enrichGO()* function was applied to identify significantly enriched GO terms across the Biological Process (BP), Cellular Component (CC), and Molecular Function (MF) categories. GO annotations were obtained from the functional annotation file AD1_TM1_T2T_ZJU_v1_genes2GO.xlsx.gz, provided by the CottonGen database, which contains GO terms assigned via InterProScan for the *Gossypium hirsutum* (AD1) ‘TM-1’ T2T genome JZU_v1.0 (Yan et al., 2025). The enrichment analysis was conducted using the hypergeometric test with the following parameters: pvalueCutoff = 0.05, pAdjustMethod = “BH” (Benjamini-Hochberg), qvalueCutoff = 0.05, and ont = “ALL”. GO terms with a false discovery rate (FDR) < 0.05 were considered significantly enriched. Additionally, to characterize gene expression change patterns over time, we carried out clustering analysis genes in roots. Using the Mfuzz time-series clustering method (R package ClusterGvis v 0.1.3, https://github.com/junjunlab/ClusterGVis), genes with similar expression dynamics across 0 h, 12 h, 24 h, and 48 h were grouped into clusters, and the expression trend of each cluster was analyzed. GO enrichment analysis was then performed for each gene cluster to reveal the main functions and pathways associated with genes of different expression patterns.

### 2.4 Weighted gene co-expression network analysis

Weighted gene co-expression network analysis (WGCNA) was employed to form gene co-expression networks (Langfelder and Horvath, 2008). The expression matrix was processed initially to remove lowly and constitutively expressed genes in the majority of samples to minimize noise in the network. Using the WGCNA R package v1.7.3, a Pearson correlation matrix was calculated for all gene pairs. An appropriate soft-thresholding power β was chosen to transform the correlation matrix into an adjacency matrix, approximately scale-free. A topological overlap matrix (TOM) was then computed from the adjacency matrix, and hierarchical clustering was performed to group genes with high co-expression into modules. After initial modules were identified via dynamic tree cutting, modules with similar expression profiles were merged to obtain the final set of co-expression modules. For each module, the module eigengene was calculated to represent the overall expression level of that module in each sample. Module eigengenes were correlated with sample phenotypic data to identify modules significantly associated with specific traits. In this study, infection time point (0, 12, 24, 48 h) was treated as a numeric trait and tissue type (root or leaf) as a categorical trait for correlation analysis, and we focused on modules highly correlated with the “infection time”. Additionally, each gene’s intramodular connectivity and module membership value was calculated; genes with the highest connectivity and significant module membership were selected as candidate hub genes for their modules.

### 2.5 Key genes selected by machine learning

To further identify key genes potentially involved in disease resistance, we employed a machine learning-based feature selection framework. A set of candidate genes was initially compiled based on differential expression analysis, expression pattern clustering (specifically, Cluster C3), and weighted gene co-expression network analysis (WGCNA). As the primary objective of this study was to identify stable and reproducible gene markers, rather than to evaluate model generalization performance, we adopted a bootstrap resampling strategy instead of a conventional train-test split. This approach enhances the robustness of feature selection and mitigates the variability and bias introduced by single-split partitioning.

We conducted 50 iterations of bootstrap sampling with replacement from the full dataset. In each iteration, a new training set equal in size to the original dataset was generated, and gene expression values were standardized using *StandardScaler*. Three machine learning algorithms implemented in Python’s scikit-learn library (v1.5.2) were then applied to independently rank feature importance: 1) LASSO regression with L1 regularization was applied using 3-fold internal cross-validation to determine the optimal penalty coefficient (λ). Genes with non-zero regression coefficients were considered important. 2) Random Forest models were constructed using 100 trees and a maximum depth of 4. Feature importance was calculated based on the Gini impurity index, and the top-ranking genes were selected. 3) SVM with a linear kernel was used to evaluate the absolute values of model coefficients, and genes with the largest weights were selected. For each model and iteration, the top 20 genes were recorded. After 50 iterations, a consensus score for each gene was calculated based on its frequency of selection across all models. Genes repeatedly selected by multiple algorithms were defined as core candidate genes.

### 2.6 qRT-PCR validation

To confirm the reliability of transcriptome data, quantitative real-time PCR (qRT-PCR) analysis was carried out with a subset of the identified key genes. Specific primers according to the sequences of genes determined by RNA-seq were designed using Primer3 (**Table 1**). Total RNA was isolated as described in RNA-seq, and then reverse-transcribed to cDNA. The internal reference gene was *GhUBQ* (ubiquitin), and both the target gene and reference were amplified as well. qRT-PCR then was implemented using SYBR Green I in real-time PCR (ABI7500, Thermofisher Scientific) with three technical replicates of every sample. The program of qRT-PCR was of a two-step nature: an initial denaturation at 95 °C, and then 40 cycles of amplification cycling between 95 °C and 60 °C. Post-amplification, melt curve analysis was done to verify primers’ specificity. The relative amounts of treatment samples with respect to 0 h were calculated by using the 2^^-ΔΔ^Ct method, and these values were compared to relative changes in expression (log_2_ fold change) measured by RNA-seq. Pearson correlation analysis was employed to assess similarity between qRT-PCR results and transcriptome data.

**Table 1:** Primers used for qRT-PCR analysis.

Gene ID	Forward Primer (5’→3’)	Reverse Primer (5’→3’)
GhChrA04G1295.1	ATGGCTGCTTCATCATCATCTG	TCAAGACAGGAATCCGTCCA
GhChrD05G0160.1	TCGGATCGGTAAAGGAGGGT	GGACGACGGGAGGTCAAAAT
GhChrA11G1796.1	AAGCCGCGACCAACAATTTC	AGCGTCTCACAGCAACAATG
GhChrD10G2704.1	AACTCCAGCAATGGCAAAGC	AACAAAAGCAAGGCCATGGC
GhChrA01G1099.1	ACATTGCAAGGCCAATCCAC	AGGTCCCAATTTTGCCAAGC
GhChrD12G2803.1	ACAGCTAAGGGTGCATTTGG	ACATTCTGTTGTGGCTGTCC
GhChrA10G0139.1	TAGAAGTGGAGAGCTCGGATAC	AATCAGCCAAAGTCCTTCCG
GhChrA11G0187.1	TTAAACGCCGGAAACACACG	TCCGATCAACCGCGAAAATC
GhChrA12G2635.1	AAGCATGCCGCATTCATGAC	ACCTTTTCAGGCCATGTTGC
GhChrA13G1329.1	TGCCATCTCATTTGCAACGG	AAGCAGTCCATTTGCCATCC
GhChrA01G0691.1	TGGTGGGAAAGATTGCTTGC	ACGCGAGGTTGATGAATTCG

## 3 Results

### 3.1 Response patterns of leaves and roots after *V. dahliae* infection

**Figure 1:**
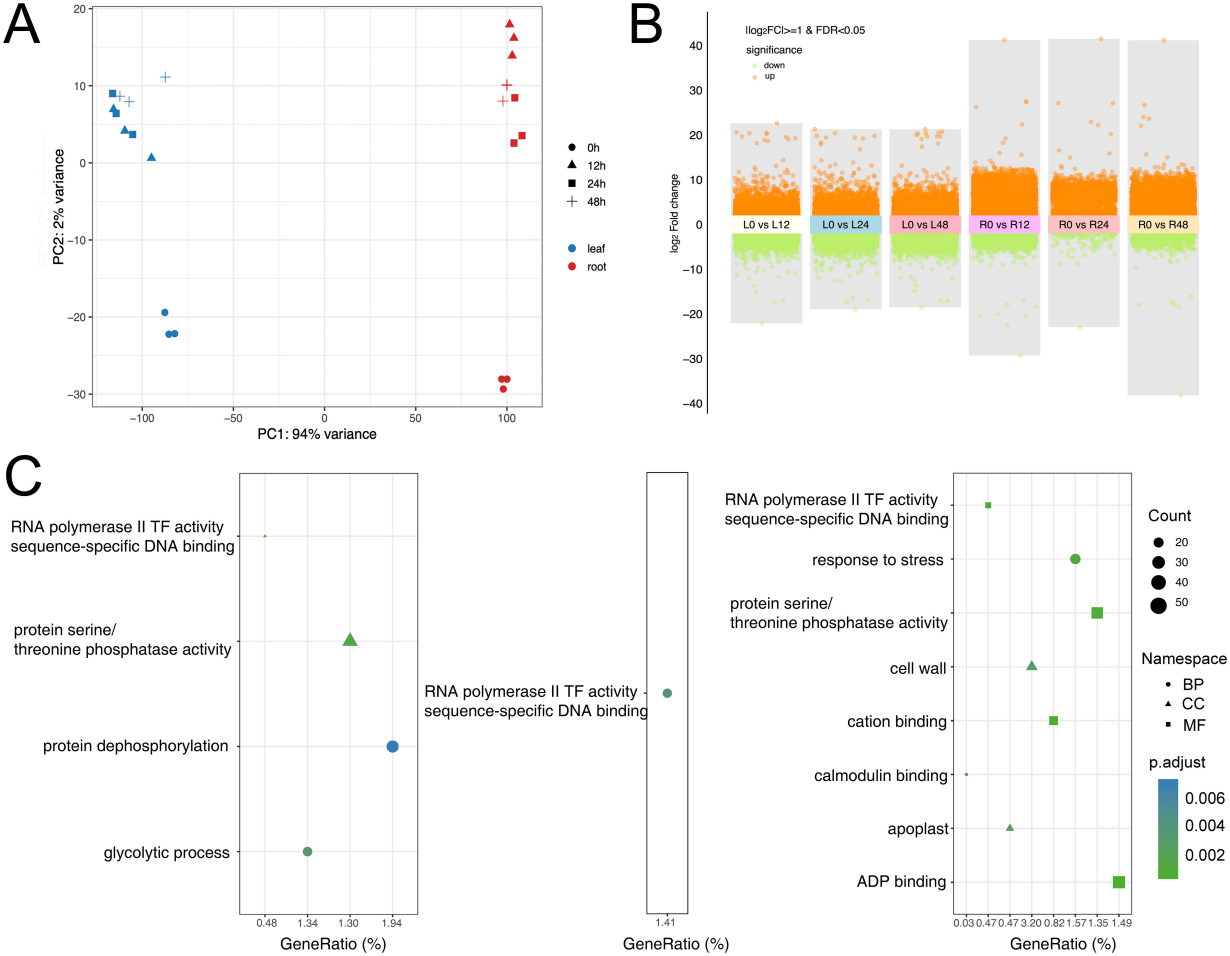
Transcriptomic responses of *G. hirsutum* to *V. dahliae* inoculation. **(A)** PCA and cluster analysis of transcriptomic data from leaf and root samples at different time points post *V. dahliae* inoculation; **(B)** DEGs in leaf and root tissues at various time points after *V. dahliae* inoculation; **(C)** GO enrichment analysis of DEGs in *G. hirsutum* roots at three time points post-inoculation compared to 0h (from left to right: 0h vs. 12h, 0h vs. 24h, and 0h vs. 48h).

We carried out RNA-seq of leaf and root samples at 0 h, 12 h, 24 h, and 48 h post-inoculation. Sequencing yielded high-quality data with consistently high mapping rates and low levels of multi-mapped reads across all samples (**Supplementary Table S1**), supporting the reliability of downstream analyses. We subsequently conducted principal component analysis (PCA), DEG identification, and GO enrichment analysis (**Figure 1**). The PCA results showed high repeatability among biological replicates within each treatment and clear separation between leaf and root samples (**Figure 1A**). This indicates that *V. dahliae* infection induces tissue-specific transcriptional responses, and that roots and leaves differ markedly in pathogen recognition and signal transduction processes. DEGs analysis revealed distinct temporal and tissue-specific patterns in *G. hirsutum* response to pathogen infection. Notably, roots exhibited much more dramatic gene expression changes: in the comparison of 0 h vs. 12 h, there were 5,128 upregulated genes and 3,757 downregulated genes in roots, far more than in leaves at the same time (2,761 upregulated and 765 downregulated). This finding suggests that as the primary infection site, roots initiate the immune response earlier and more strongly. As the disease progressed, roots continued to sustain a higher level of gene expression changes at 24 h and 48 h (**Figure 1B**). Therefore, subsequent analyses focused on the root samples. GO enrichment analysis of root DEGs at each time point revealed that such genes were enriched in many defence-related biological processes (**Figure 1C**). DEGs at 12 h post-inoculation were significantly enriched in terms related to ‘RNA polymerase II transcription factor activity’, ‘sequence-specific DNA binding’, ‘protein serine/threonine phosphatase activity’, and ‘glycolytic process’. This indicates that at the initial infection period, cotton roots responded quickly by activating transcriptional regulators, signal transduction factors, and energy metabolisms. By 48 h, DEGs became enriched in terms such as “calmodulin binding”, “cation binding”, “response to stimulus/stress”, “cell wall”, and “apoplast”, indicating that at later stages the roots may enhance defense by modulating calcium signaling pathways, the extracellular environment, and cell wall structure.

### 3.2 Gene expression pattern analysis

**Figure 2:**
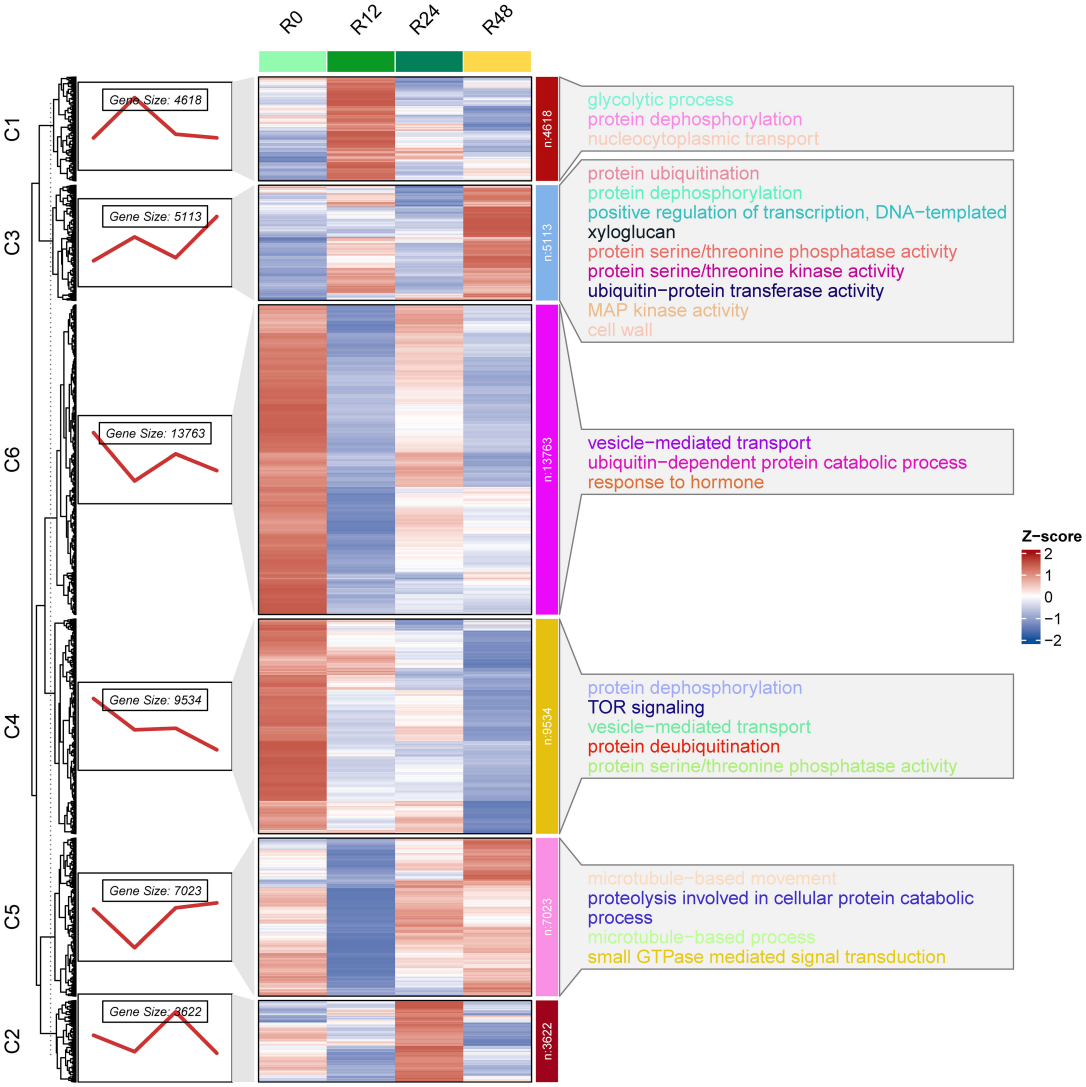
Temporal expression patterns and GO enrichment analysis of co-expressed gene clusters in *G. hirsutum* roots in response to *V. dahliae* infection. Gene expression profiles were obtained from cotton roots at 0 h, 12 h, 24 h, and 48 h after *V. dahliae* infection, and genes were grouped into six co-expressed clusters (C1–C6). The left panel shows the dynamic expression trends of each cluster across the four time points. The heatmaps represent the expression levels within each cluster (Z-score), where red indicates high expression and blue indicates low expression. The right panel displays the GO enrichment results for each cluster.

To further dissect the temporal changes in gene expression in *G. hirsutum* roots during *V. dahliae* infection, we performed clustering analysis on all gene expression profiles from the root samples. Based on expression changes at 0, 12, 24, and 48 h, the root DEGs were grouped into 6 co-expression clusters (C1–C6) (**Figure 2**, left panel). Different clusters showed distinct temporal expression patterns. For example, C1 (4,618 genes) represents genes that were rapidly upregulated at the early stage of infection and then declined, being strongly induced at 12 h and then gradually falling off by 24-48 h. Clusters C3 (5,113 genes) and C5 (7,023 genes) showed a trend of sustained or delayed upregulation, reaching their highest expression at 48 h. These patterns suggest that cotton roots harbor two major groups of responsive genes: early transient response genes and later sustained response genes. GO enrichment analysis was performed for each gene cluster to explore the functional categories associated with the different expression patterns (**Figure 2**, right panel). The results showed that the functions enriched in each cluster corresponded to their expression timing. Gene clusters with high expression at early stages (such as C1 and C2) were significantly enriched in functions related to transcriptional regulation and signal transduction, including transcription factor activity and protein kinase and phosphatase activity. In contrast, clusters that were continuously or later upregulated (C3 and C5) were enriched in metabolic and defense response processes. Of particular note, C3 genes were notably enriched in some significant biological processes related to defense, such as ‘defense response’, ‘reactive oxygen metabolic process’, ‘secondary metabolic process’, and ‘hormone-mediated signaling pathway’. This suggests that during the infection process, C3 genes will be continuously triggered and coordinate intricate defense mechanism in cotton, such as oxidative burst, synthesis of antimicrobial compounds, and hormone signaling, to effectively block further invasion and spread of the pathogen.

### 3.3 WGCNA analysis

**Figure 3:**
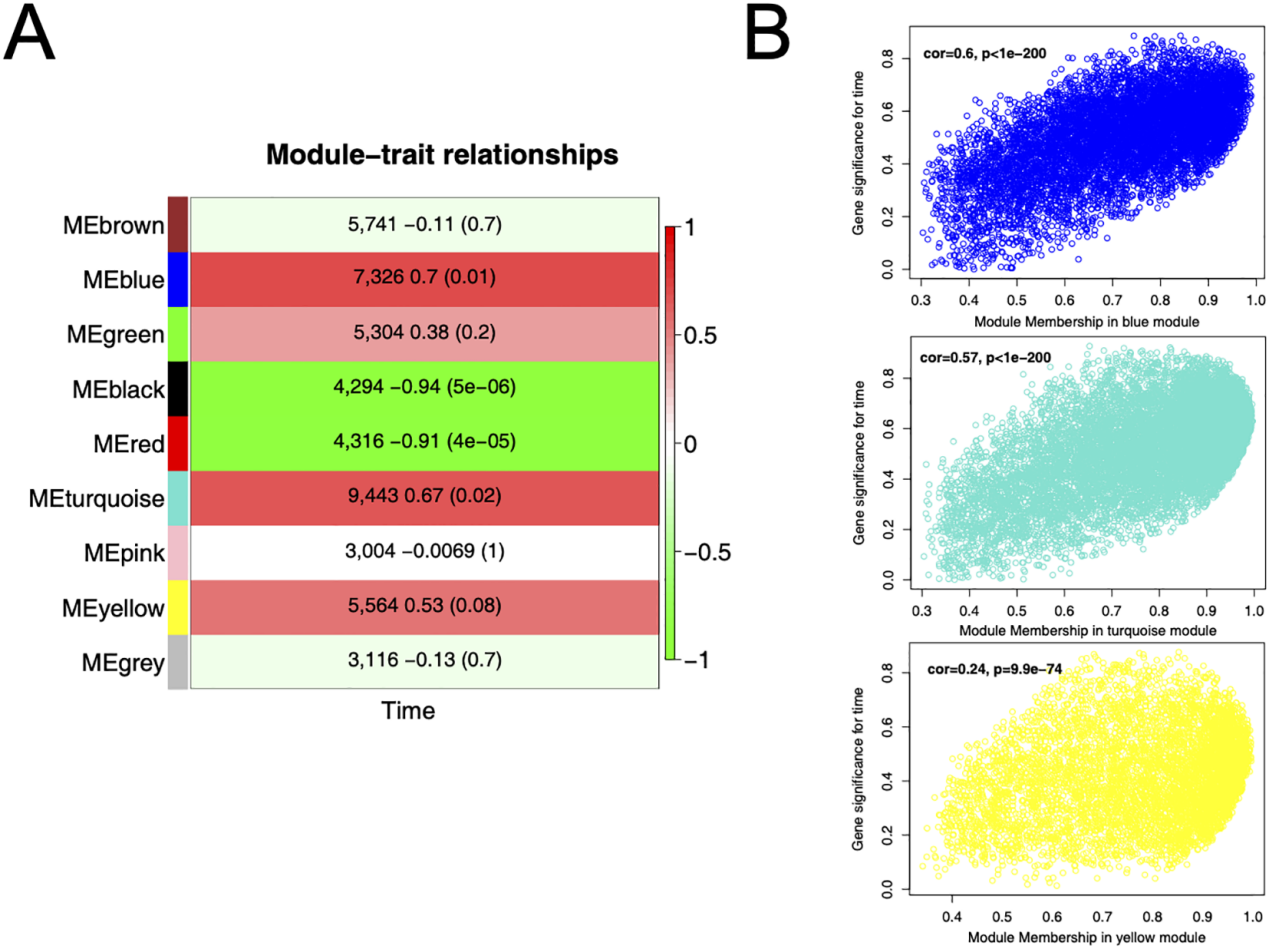
WGCNA of cotton in response to *V. dahliae* infection. **(A)** Heatmap shows the correlation between each module and the time trait. The numbers in the boxes indicate Pearson correlation coefficients, with the corresponding *p*-values shown in parentheses. The black and red modules exhibit significant correlations with time. **(B)** Correlation analysis between module membership and gene significance for the 3 time-associated modules (blue, turquoise, and yellow), with correlation coefficients of 0.6 (*p* < 1e−200), 0.57 (*p* < 1e−200), and 0.24 (*p* = 9.9e−74), respectively.

To analyze the coordinated expression of resistance genes from a global network perspective, we conducted WGCNA on the transcriptome data. A total of 16 co-expression modules were detected based on the expression profiles of all samples, with each module labeled by a different color. We focused on modules that were significantly correlated with the infection time course, in order to identify gene groups exhibiting specific patterns as the disease progressed. Correlation analysis revealed that the blue module, turquoise module, and yellow module had module eigengenes strongly positively correlated with time (Pearson correlation coefficients 0.60, 0.57, and 0.24, respectively; *p* values all highly significant, **Figure 3A**), meaning the genes in these modules showed continuously increasing expression as infection time progressed. These results indicate that the positively time-correlated modules are rich in defense-related genes that are activated progressively as the disease develops.

To further understand the biological functions of the key modules, we examined the functional composition and representative genes of the modules that were positively correlated with time. The blue module contains 7,326 genes and is enriched for many known disease resistance-related genes, including various signaling components and defense enzymes. The turquoise module has 9,443 genes and is significantly enriched in secondary metabolism and stress response functions. The yellow module has relatively fewer genes (5,564) but is rich in diverse transcriptional regulators. Notably, each of these modules contains several genes that were already identified as significantly upregulated in the differential expression and clustering analyses, further underscoring their importance in cotton’s defense response. We calculated intramodular connectivity for each gene and identified hub genes in each module. For example, in the blue module, the genes with the highest connectivity included those encoding receptor-like protein kinases and pathogenesis-related proteins; in the turquoise module, many of the hub genes were metabolic enzymes and signaling molecules; and the yellow module’s hub genes were predominantly transcription factors. These hub genes likely play central roles in the function of their respective modules, driving the coordinated expression of other genes in the module. The characteristics of these modules reflect the major defense activities of cotton at different times, such as a continuous enhancement of defense metabolism and signal transduction throughout the infection.

### 3.4 Machine learning analysis of key genes

By applying the machine learning feature selection, we obtained a high-confidence list of core resistance genes. In total, 15 genes were consistently selected by all three algorithms (**Table 2**). These genes all showed high importance in the machine learning models, indicating that their expression changes are closely associated with cotton’s response to *V. dahliae* infection.

**Table 2:** LASSO-RF-SVM hub genes and their WGCNA network modules in cotton.

Gene ID	Function	LASSO	RF	SVM	Models Selected	Module in WGCNA
GhChrA05G3082.1	Unknown	26	8	8	LASSO, RF, SVM	yellow
GhChrA01G0490.1	Receptor-like protein 6	16	4	21	LASSO, RF, SVM	turquoise
GhChrD11G1349.1	Gibberellin receptor GID1B	2	1	37	LASSO, RF, SVM	yellow
GhChrD11G3762.1	disease resistance protein RPP2B-like	0	5	34	RF, SVM	turquoise
GhChrD04G1036.1	UDP-glucuronate 4-epimerase 1	30	1	0	LASSO, RF	turquoise
GhChrA04G0025.1	chaperone protein dnaJ 11	10	7	13	LASSO, RF, SVM	turquoise
GhChrD05G0556.1	CBL-interacting kinase 6	1	1	28	LASSO, RF, SVM	yellow
GhChrA09G0965.1	Unknown	2	5	22	LASSO, RF, SVM	/
GhChrA12G3377.1	Calmodulin-binding protein 60 A	2	1	23	LASSO, RF, SVM	turquoise
GhChrA09G0007.1	Unknown	9	4	13	LASSO, RF, SVM	turquoise
GhChrA10G2543.1	Pyruvate kinase 1	15	6	4	LASSO, RF, SVM	green
GhChrA03G1605.1	Unknown	17	3	5	LASSO, RF, SVM	black
GhChrA05G0662.1	Coatomer subunit beta-1	2	4	18	LASSO, RF, SVM	blue
GhChrA02G0724.1	Scarecrow-like protein 1	17	3	3	LASSO, RF, SVM	brown
GhChrD05G1517.1	E3 ubiquitin-protein ligase RSL1	0	0	21	SVM	turquoise
GhChrD03G2069.1	Regulator of nonsense transcripts 1	12	8	0	LASSO, RF	/
GhChrD02G2071.1	Unknown	1	6	13	LASSO, RF, SVM	turquoise
GhChrD05G0070.1	xyloglucan endotransglucosylase/hydrolase protein 22	18	2	0	LASSO, RF	turquoise
GhChrA03G0198.1	flavonoid 3’-monooxygenase	7	1	11	LASSO, RF, SVM	blue
GhChrA11G3574.1	Unknown	1	8	10	LASSO, RF, SVM	/

The functional annotations of the core genes listed in **Table 1** show that they encompass multiple defense-related categories, including signal perception, hormone signaling pathways, transcriptional regulation, and metabolism. This suggests that cotton’s resistance to *V. dahliae* is achieved through multi-layered regulatory processes. The core genes include receptor protein genes (e.g., *GhRLP6*, encoding receptor-like protein 6), resistance protein genes (e.g., *RPP2B*-like disease resistance protein), calcium signaling-related genes (e.g., *GhCIPK6*, encoding CBL-interacting protein kinase 6; *GhCBP60A*, calmodulin-binding protein 60 A), hormone signaling genes (e.g., *GhGID1B*, a gibberellin receptor), transcription factor genes (e.g., *GhSCL1*, a member of the GRAS family), as well as various genes involved in metabolism and protein processing (such as UDP-glucuronate 4-epimerase 1, a chaperone DnaJ11 protein, etc.). These genes cover the key layers of plant defense-pathogen recognition, signal transduction, transcriptional reprogramming, and execution of defense-implying that they collectively contribute to cotton’s resistance through coordinated action.

### 3.5 Co-expression network analysis of key genes

**Figure 4:**
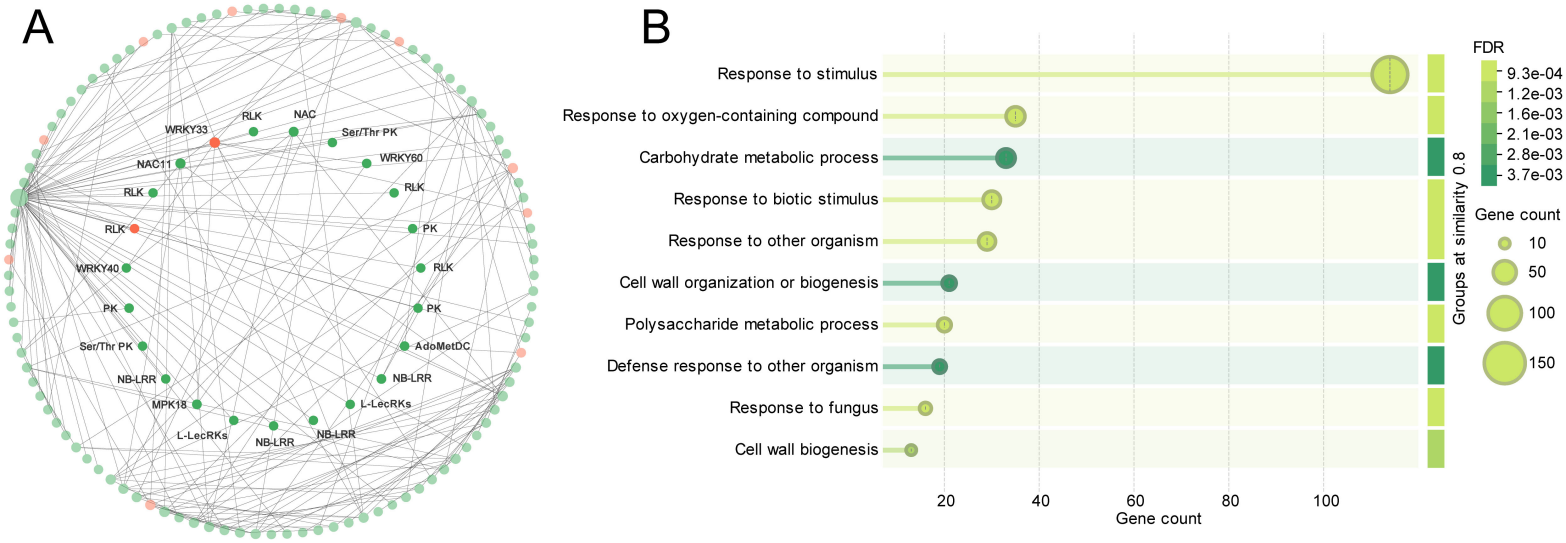
Regulatory network and GO enrichment analysis of key module genes in cotton roots in response to *V. dahliae* infection. **(A)** Gene regulatory network of hub genes within key co-expression modules. Each node represents a gene, with node size indicating its connectivity in the network. Node colors denote gene selection sources: red indicates key genes identified by machine learning, and green marks genes jointly identified by DEGs, Cluster C3 from expression pattern analysis, and yellow, turquoise, and blue modules from WGCNA. Key transcription factors, receptor-like kinases, and other regulators are labeled. **(B)** GO enrichment analysis of genes from the key modules. The bar chart shows the number of genes enriched in each biological process term, with color gradients indicating FDR values.

To gain deeper insight into the key regulatory genes and the defense pathways they participate in, we performed an integrated analysis combining the DEGs, the genes in cluster C3, the WGCNA module results, and the machine learning-selected core genes. We constructed a co-expression gene network of these key genes (**Figure 4A**). In this network, each node represents a key gene; node size reflects the gene’s connectivity (degree) within the network, and node color indicates the gene’s source category. Red nodes represent core hub genes identified by all three machine learning methods (LASSO, RF, SVM), while green nodes represent important resistance-related genes that were prominently featured in DEGs and clustering analysis (C3 cluster), as well as in the WGCNA analysis (genes from the yellow, turquoise, and blue modules).

Network analysis revealed that these crucial genes represent multiple general plant disease-resistance functional groups, and notably include NBS-LRR class resistance proteins, receptor-like kinases (RLKs, of which L-type lectin receptor kinases, L-LecRKs, are included), transcription factors (such as WRKY33, WRKY40, WRKY60, and NAC family members like NAC11), and signaling protein kinases (e.g., PKs, Ser/Thr protein kinases, MPK18), among other core regulators. Specifically, WRKY transcription factors WRKY33, WRKY40, and WRKY60 have high centrality within the network, and it can be speculated that they play significant regulatory functions in cotton infection response by directly regulating many downstream gene expressions. Moreover, several NBS-LRR genes, traditional plant R genes, occupy significant network positions and suggest that effector-triggered immunity (ETI) would be crucial to cotton Verticillium wilt resistance response. The receptor-like kinases (RLKs) and L-LecRKs in the network could be involved in activating initial defense response by perceiving pathogen-associated molecular patterns (PAMPs).

To further identify biological processes involved in these pivotal genes, we carried out GO enrichment analysis of this group of genes (**Figure 4B**). The analysis revealed that these pivotal genes are enriched predominantly in significant terms like ‘response to stimulus’, ‘response to oxygen-containing compound’, ‘carbohydrate metabolic process’, ‘response to biotic stimulus’, ‘cell wall organization or biogenesis’, and ‘defense response to other organism’. The enrichment of ‘defense response’ and ‘cell wall organization or biogenesis’ suggests that at mid-to-late infection stages of cotton resistance, the plant presumably organizes its cell wall structure and controls deposition of cell wall compounds to physically block spread of the pathogen and at the same time to synthesize and secrete antimicrobial secondary metabolites to defend chemically.

### 3.6 qRT-PCR validation of key genes

**Figure 5:**
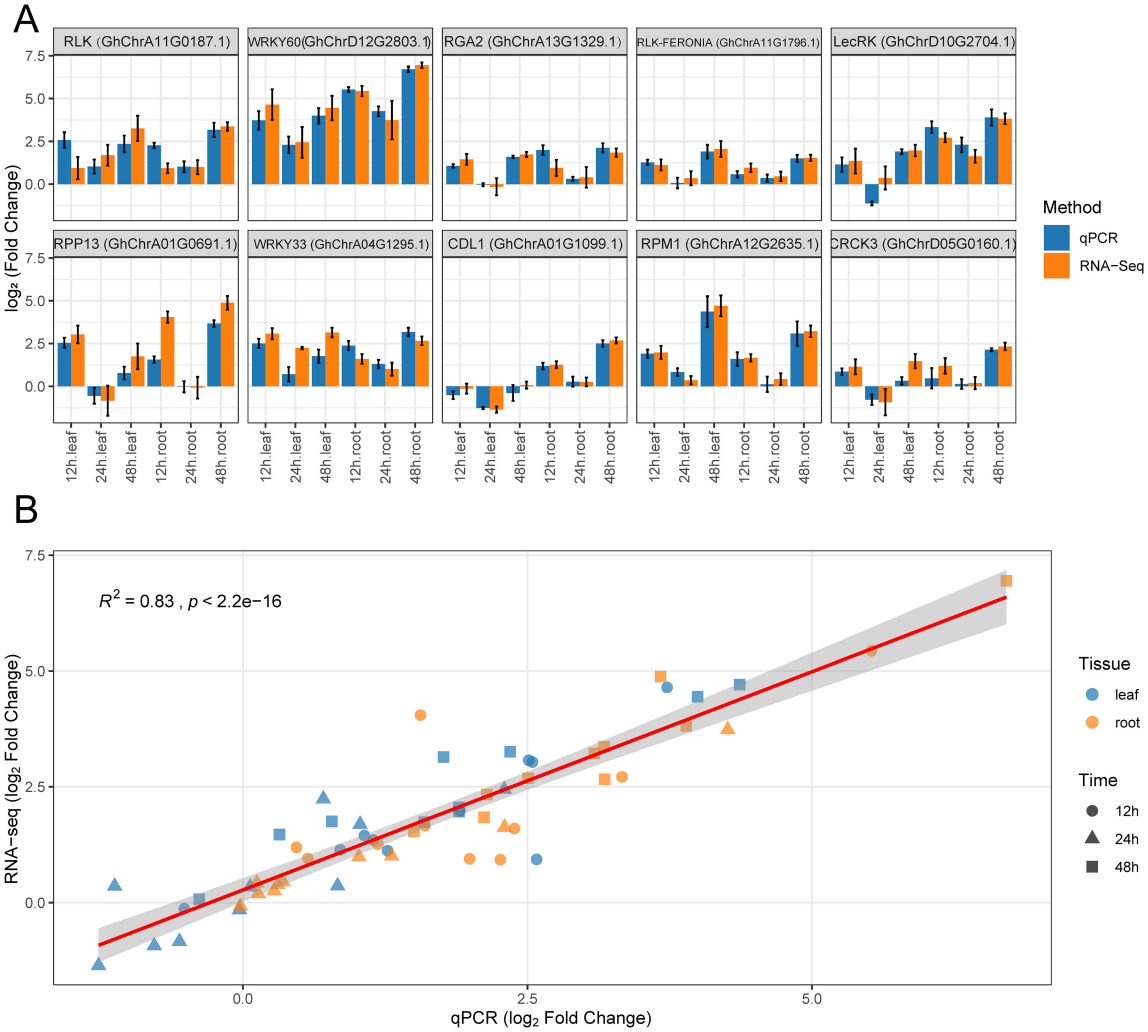
Expression patterns of key genes validated by qRT-PCR. **(A)** Log_2_ fold changes of 10 key genes at 12 h, 24 h, and 48 h in leaf and root tissues, relative to 0 h. Blue bars represent qPCR results, and orange bars represent RNA-seq data, with error bars indicating standard error. **(B)** Correlation analysis between qRT-PCR and RNA-seq results. Different shapes represent different time points, and different colors represent tissue types. The red regression line indicates a strong positive correlation between the two methods (R² = 0.83, *p* < 2.2e^−16^).

To verify the reliability of the expression patterns for key genes observed in the transcriptome data, we selected 10 key genes for qRT-PCR analysis. These genes included a representative set of different functional categories: a receptor gene (*GhRLP6*), a kinase gene (*GhCIPK6*), a resistance protein gene (*GhRPP2B*-like), a transcription factor gene (*GhSCL1*), and several enzyme genes (*GhPK1* encoding pyruvate kinase 1, GhF3’H encoding flavonoid 3’-monooxygenase, etc.). The analysis showed that the expression changes of these genes measured by qRT-PCR were highly consistent with the RNA-seq data (**Figure 5A**). For example, the expression of *GhRLP6* in roots was about 5-fold higher at 12 h compared to 0 h, continued to rise at 24 h, and peaked at 48 h. The corresponding RNA-seq results showed log_2_ fold changes of 2.3, 3.1, and 3.6 at those time points, which closely matched the qRT-PCR quantification. Similarly, genes such as *GhCIPK6* and *GhCBP60A* showed strong induction in roots and relatively lower changes in leaves, and these patterns were well validated by qRT-PCR. To quantitatively assess the consistency between the two methods, we performed a correlation analysis of the expression values for all the validated genes across different times and tissues (**Figure 5B**). The results showed a significant positive correlation between the relative expression levels measured by qRT-PCR and the TPM changes from RNA-seq (Pearson R² = 0.83, *p* < 2.2×10^−16^), indicating that the transcriptome data are highly reliable.

## 4 Discussion

In this study, a time-course transcriptome analysis, comprehensively revealed the dynamic defense response characteristics of cotton under *V. dahliae* infection. The results indicate that roots and leaves play different roles and operate on different timelines in the resistance response. Roots, as the frontline of pathogen entry, quickly detect the pathogen and activate a large number of defense-related genes at the early stage of infection, whereas the leaves mainly initiate defense responses at later stages upon receiving systemic signals. This pattern is consistent with the concept of local immunity and systemic acquired resistance (SAR) in plants (Wang et al., 2025). At 12 h post-inoculation, cotton roots already show a significant transcriptional response (5,128 upregulated genes, 3,757 downregulated genes), which is markedly stronger than the response in leaves at the same time (2,761 upregulated, 765 downregulated genes, **Figure 1B**). Such tissue-specific differences in early response are also observed in other plants like tomato and *Arabidopsis* during early infection by *Verticillium* spp., where roots mount a faster and more intense initial immune response than leaves (Buhtz et al., 2015; Rich-Griffin et al., 2020). This is likely related to the roots’ direct role in sensing the pathogen and blocking its further invasion into the plant.

At 12 h after *V. dahliae* inoculation, the roots’ DEGs were significantly enriched in GO terms such as “RNA polymerase II transcription factor activity”, “sequence-specific DNA binding”, and “glycolytic process” (**Figure 1C**). This indicates that at the early stage, cotton roots are rapidly activating transcription factors and fundamental energy metabolism pathways to cope with the stress of pathogen attack. Recent studies have shown that quick activation of the glycolysis pathway not only provides sufficient energy for immune signal transduction but may also directly participate in defense signaling through metabolic intermediates (Guo et al., 2022). Therefore, the dramatic changes in gene expression at early time points likely represent a critical strategy for cotton to rapidly establish the first line of defense.

Cotton resists pathogen invasion and dissemination by fortifying its cell wall structure and enhancing the deposition of lignin and flavonoid compounds. This is particularly visible in the C3 and C5 gene clusters (5,113 and 7,023 genes, respectively), representative of later-stage sustained response genes. The clusters were highly enriched in processes such as MAPK kinase activity, ubiquitination of proteins, and reinforcement of cell walls (**Figure 2**). It is hypothesized that at mid-to-late infection stages, cotton is thought to continuously amplify defense signals by MAPK cascades and modulate sustained transcription of downstream defense genes to fortify the cell wall and stimulate secondary metabolism (Mi et al., 2024). The MAPK signaling pathway has been identified as an effective pathway in *Verticillium* infection responses in plants and is capable of modulating transcriptional activity of a series of resistance-related genes and increasing the sustained resistance of plants to the infection (Mi et al., 2024; Zhu et al., 2022). Previous studies have indicated that genetic enhancement of lignin accumulation in cotton can effectively increase resistance to *Verticillium* wilt (Hu et al., 2021; Liu et al., 2024; Xiong et al., 2021). Concurrently, naphthoquinones and flavonoids are significant antimicrobial compounds in cotton (Long et al., 2023; Zu et al., 2023). Flavonoid 3’-monooxygenase, as a pivotal enzyme in secondary metabolism, can potentially inhibit growth and invasion of hyphae of pathogens and spread by catalyzing synthesis of certain antimicrobial flavonoids and concurrently mitigate oxidative damage due to infection at the same time. The enzyme has been extensively reported to increase resistance of plants to several kinds of pathogens (Wang et al., 2021). Therefore, accumulation of lignin and flavonoid-like compounds seems to be an integral feature of subsequent-stage defence.

With WGCNA, we determined 2 significant modules significantly associated with infection process: the blue module (7,326 genes) and turquoise module (9,443 genes), both of them strongly positively correlated with infection time (**Figure 3**). Both modules were enriched with many receptor-like kinases, calcium-signaling-related genes, and disease resistance protein genes. This indicates that at mid-to-late infection stages of the pathogen, cotton probably reinforces its resistance by integrating multiple hierarchies of defense signaling pathways. Receptor-like proteins would receive firstly pathogen signal, then initiate activation of calcium signaling pathways, and these, by regulators like *GhCIPK6* and *GhCBP60A*, further amplify and perpetuate downstream defense gene expressions. Integrating the significant genes determined by machine learning (**Table 1**), we further identified the molecular players that likely function as chief components in resistance, such as receptor protein *GhRLP6*, resistance protein *GhRPP2B*-like, and calcium-signaling components *GhCIPK6* and *GhCBP60A*, and flavonoid 3’-monooxygenase as a metabolic enzyme (Kumari et al., 2022; Vishwakarma et al., 2025). We surmised that *GhRLP6* as a PR (pattern recognition receptor) sensor is likely to detect pathogen-associated molecular patterns (PAMPs) in cotton roots, as in Arabidopsis some of the RLPs stimulate plasma membrane immune complexes and stimulate broad-spectrum resistance (Zhang et al., 2025a). The *GhRPP2B*-like gene falls in the traditional NB-LRR group of R genes; it would increase pathogen effector recognition by mediating ETI (effector-triggered immunity) and induce hypersensitive response (HR) cell death and restrict pathogen spread (Chicowski et al., 2024). Also, Arabidopsis and rice plants have revealed that calcium-sensing molecules *GhCIPK6* and *GhCBP60A* induce wide-spectrum disease resistance by maintaining calcium ion flow and downstream transcriptional reprogramming, and it’s probable that they also play significant parts in cotton Verticillium wilt resistance (Vishwakarma et al., 2025; Yu et al., 2025). We speculate that *GhRLP6*, as a pattern recognition receptor (PRR), is responsible for sensing pathogen-associated molecular patterns (PAMPs) in cotton roots, similar to how in Arabidopsis certain RLPs activate plasma membrane immune complexes and induce broad-spectrum resistance (Zhang et al., 2025a). The *RPP2B*-like gene belongs to the classic NB-LRR class of R genes; it may enhance recognition of pathogen effectors by mediating effector-triggered immunity (ETI) and trigger hypersensitive response (HR) cell death, thereby limiting pathogen spread (Chicowski et al., 2024). In addition, the calcium-signaling molecules *CIPK6* and *CBP60A* have been shown in *Arabidopsis* and rice to promote broad-spectrum disease resistance by regulating calcium ion flux and downstream transcriptional reprogramming, suggesting they likely play important roles in cotton’s *Verticillium* wilt resistance as well (Vishwakarma et al., 2025; Yu et al., 2025).

## 5 Conclusion

In this study, we systematically characterized the dynamic transcriptional responses and molecular resistance mechanisms of *G. hirsutum* to *V. dahliae* infection. Our results demonstrate that roots serve as the primary site of early immune activation, rapidly initiating PAMP-triggered signaling and transcriptional reprogramming, highlighting a distinct spatial-temporal defense pattern. Through differential expression analysis, co-expression network construction, and machine learning-based screening, we identified key defense genes involving receptor-mediated pathogen recognition, calcium and hormone signaling, transcriptional regulation, and secondary metabolism. A number of pivotal genes, such as *GhRLP6*, *GhCIPK6*, *GhCBP60A*, GhSCL1, and *GhF3’H*, were identified as primary regulators of *G. hirsutum* resistance. The results show that cotton *Verticillium* wilt resistance is controlled by complicated regulatory networks and multi-level defense mechanisms. Our research not only sheds light on the novel molecular mechanism of disease resistance in cotton but provides useful gene resources for improving Verticillium wilt resistance in cotton. Future efforts focusing on functional validation of these candidate genes and elucidation of their regulatory networks will further advance molecular breeding strategies for developing resistant cotton cultivars, ensuring more sustainable cotton production.

## Data Availability

The RNA-seq data generated in this study have been deposited in the (BIG Submission, BIG Sub) under BioProject accession number PRJCA042553.

## Supplementary material

Supplementary Figure 1 Soft-thresholding and Module Detection in Cotton Gene Co-expression Network under *V. dahliae* Infection. **(A)** Soft-thresholding power selection. The left panel shows the scale-free topology fit index as a function of soft-thresholding power, used to determine an appropriate weighting parameter; the right panel displays the mean connectivity corresponding to each power, reflecting the degree of gene connectivity. **(B)** Hierarchical clustering dendrogram of gene co-expression modules based on topological overlap, with different colors representing distinct modules.
