# Supplementary figures and images for: Integrative identification of key genes governing Verticillium wilt resistance in Gossypium hirsutum using machine learning and WGCNA
Source: Front Plant Sci. 2025 Jul 28;16:1621604. doi: 10.3389/fpls.2025.1621604 (PMC12336154; doi:10.3389/fpls.2025.1621604)

# A

### Scale independence

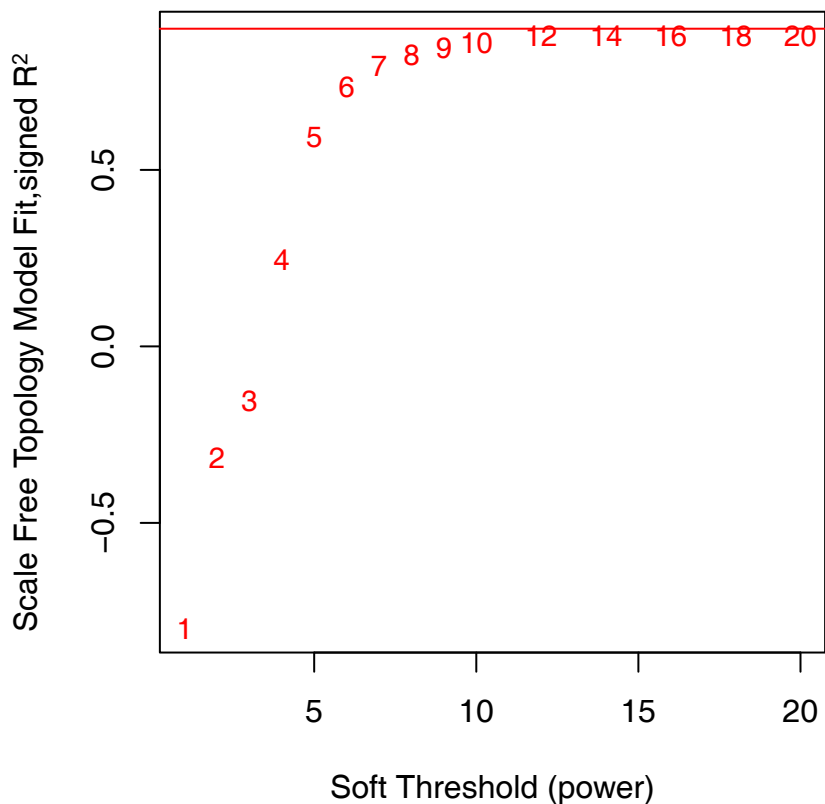

### Mean connectivity

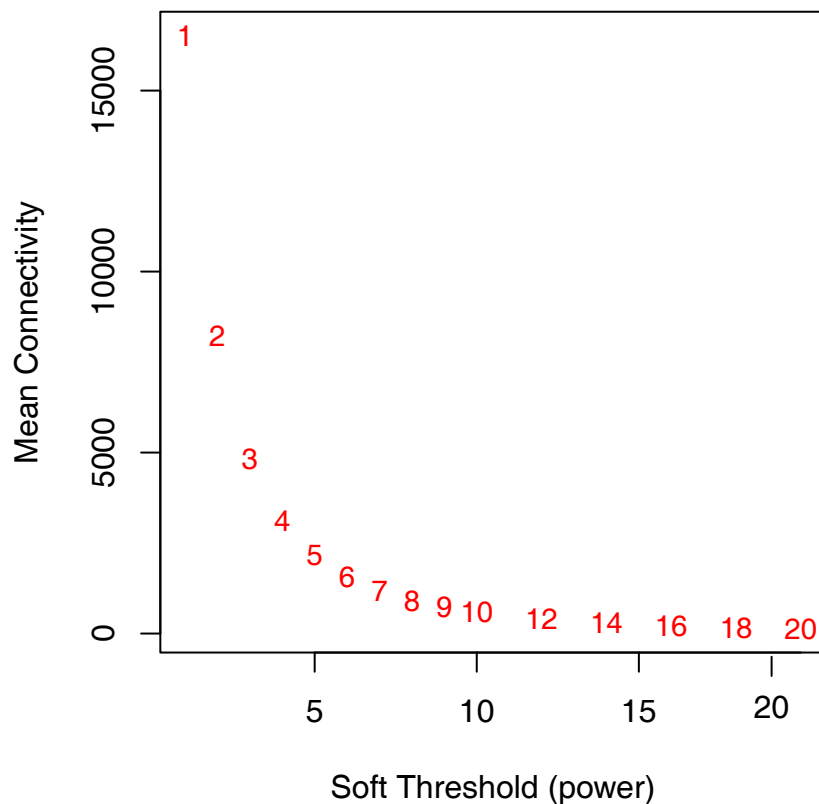

# B

### Cluster Dendrogram

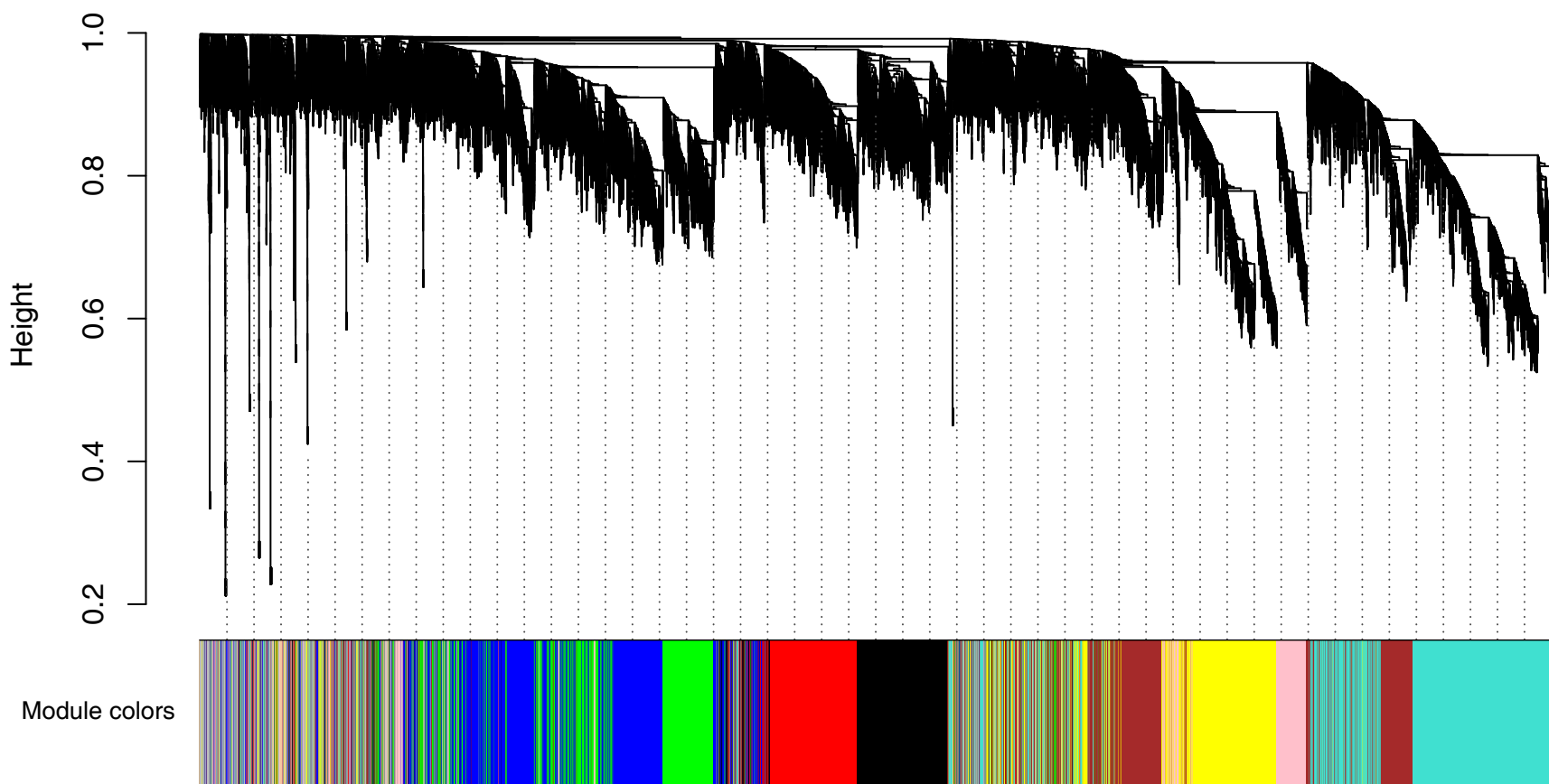

Supplement: Supplementary Figure 1 — Soft-thresholding and Module Detection in Cotton Gene Co-expression Network under V. dahliae Infection. (A) Soft-thresholding power selection. The left panel shows the scale-free topology fit index as a function of soft-thresholding power, used to determine an appropriate weighting parameter; the right panel displays the mean connectivity corresponding to each power, reflecting the degree of gene connectivity. (B) Hierarchical clustering dendrogram of gene co-expression modules based on topological overlap, with different colors representing distinct modules. [file Image1.pdf]
