# Supplementary material for: Integrative identification of key genes governing Verticillium wilt resistance in Gossypium hirsutum using machine learning and WGCNA
Source: Front Plant Sci. 2025 Jul 28;16:1621604. doi: 10.3389/fpls.2025.1621604 (PMC12336154; doi:10.3389/fpls.2025.1621604)
Supplement: Supplementary file 2 [file Table1.docx]

Supplementary Table S1. RNA-seq Mapping and Read Quality Summary

| Sample | Clean Reads | Mapped (%) | Uniquely Mapped (%) | Multi-mapped (%) |
| --- | --- | --- | --- | --- |
| leaf0h_1 | 19738241 | 97.67 | 86.32 | 4.41 |
| leaf0h_2 | 19023575 | 98.01 | 86.52 | 4.54 |
| leaf0h_3 | 20425261 | 98.01 | 87.83 | 4.09 |
| leaf12h_1 | 23786131 | 97.83 | 86.57 | 4.46 |
| leaf12h_2 | 20968373 | 97.69 | 85.89 | 4.65 |
| leaf12h_3 | 20375366 | 97.54 | 86.41 | 4.43 |
| leaf24h_1 | 19346287 | 98.12 | 86.2 | 4.63 |
| leaf24h_2 | 20834407 | 97.56 | 86.15 | 4.83 |
| leaf24h_3 | 20146619 | 97.65 | 81.5 | 5.34 |
| leaf48h_1 | 21016778 | 91.88 | 81.75 | 4.2 |
| leaf48h_2 | 21881538 | 97.55 | 84.45 | 4.85 |
| leaf48h_3 | 20971940 | 97.43 | 86.14 | 4.46 |
| root0h_1 | 20822420 | 97.45 | 86.53 | 4.46 |
| root0h_2 | 22203190 | 96.64 | 83.48 | 4.94 |
| root0h_3 | 21834983 | 97.24 | 83.64 | 5.01 |
| root12h_1 | 20719459 | 94.96 | 82.79 | 4.65 |
| root12h_2 | 22604597 | 95.22 | 81.19 | 5.01 |
| root12h_3 | 21459730 | 95.87 | 83.24 | 4.77 |
| root24h_1 | 26325183 | 95.97 | 80.14 | 5.57 |
| root24h_2 | 31721719 | 96.17 | 81.17 | 5.53 |
| root24h_3 | 23622991 | 96.34 | 83.96 | 4.69 |
| root48h_1 | 20038044 | 95.89 | 82.47 | 5.08 |
| root48h_2 | 19938586 | 92.74 | 79.78 | 5.02 |
| root48h_3 | 19659880 | 93.83 | 81.52 | 4.78 |
