# Supplementary material for: Integrative identification of key genes governing Verticillium wilt resistance in Gossypium hirsutum using machine learning and WGCNA
Source: Front Plant Sci. 2025 Jul 28;16:1621604. doi: 10.3389/fpls.2025.1621604 (PMC12336154; doi:10.3389/fpls.2025.1621604)
Supplement: Supplementary file 3 [file Table2.docx]

Supplementary Table 2. Average Classification Performance of LASSO, Random Forest, and SVM Across 50 Bootstrap Iterations

| Model | Accuracy | Precision | Recall | F1-score |
| --- | --- | --- | --- | --- |
| LASSO | 0.853 | 0.732 | 0.662 | 0.698 |
| RF | 0.927 | 0.767 | 0.725 | 0.742 |
| SVM | 0.930 | 0.798 | 0.801 | 0.796 |
